# The Positioning Method of Pulmonary Nodules in Thoracoscopic Surgery Based on CT Simulation Positioning System for Radiotherapy

**DOI:** 10.5761/atcs.oa.24-00148

**Published:** 2025-04-04

**Authors:** Jiandong Hong, Taobo Luo, Yan Zhang, Ying Chen, Yang Pan, Haoting Xu, Jian Zeng

**Affiliations:** 1 School of Medicine, Shaoxing University, Shaoxing, Zhejiang, China; 2 Department of Pulmonary Surgery, Zhejiang Cancer Hospital, Hangzhou, Zhejiang, China; 3 Wenzhou Medical University, Wenzhou, Zhejiang, China; 4 Zhejiang Key Laboratory of Diagnosis and Treatment Technology on Thoracic Oncology (Lung and Esophagus), Zhejiang Cancer Hospital, Hangzhou, Zhejiang, China; 5 Department of Radiation Oncology, Zhejiang Key Laboratory of Radiation Oncology, Zhejiang Cancer Hospital, Hangzhou, Zhejiang, China

**Keywords:** CT simulation positioning system of radiotherapy, wedge resection, surface positioning, intraoperative puncture positioning, small pulmonary nodules

## Abstract

**Purpose:** The application of wedge resection in thoracoscopic surgery is becoming more and more widely prevalent. However, achieving precise intraoperative positioning of the pulmonary nodules still poses challenges. This study proposed a method for surface positioning using a computed tomography (CT) simulation positioning system in the radiation physics room.

**Methods:** After screening patients, the level of nodules was located under the CT simulation positioning system, and the pleural projection point of the nodule and the closest surface puncture point from this point to the body surface were determined by the laser positioning system. During the operation, a needle was inserted at a predetermined angle at the puncture point, leaving a pinhole in the visceral pleura. Finally, the distance between the true pleural projection point of the nodule and the pinhole was measured on the specimen.

**Results:** The success rate of our positioning method was 97.2%. The average distance between the puncture pinhole location and the actual pleural projection point of the nodule was 8.1 mm. No related complications occurred during the perioperative period.

**Conclusion:** The new method of preoperative surface positioning and intraoperative lung positioning through puncture has a high success rate, good positioning accuracy, and good safety, which is worthy of clinical application.

## Introduction

With the increasing popularity of routine computed tomography (CT) scans and promotion of the application of thin-slice CT equipment, the detection rate of pulmonary nodules has rapidly increased. Among them, pure ground glass opacities (GGOs) and mixed GGOs had a higher malignant probability, wherein most of them were early lung cancer with low-grade malignancy.^1–3)^ According to the National Comprehensive Cancer Network (NCCN) guidelines, the American College of Chest Physicians (ACCP) guidelines, and conclusions of the Japan Clinical Oncology Group (JCOG) series studies, sublobar resection was recommended as the first choice for GGOs with surgical indications, because it had a lesser impact on the pulmonary function.^4)^ For patients with peripheral GGOs, wedge resection was often the preferred surgical method.^5)^

For successful wedge resection, the most important thing was to accurately find the location of the lesion. The traditional method was through intraoperative direct vision and finger touch. Most nodules were not on the pleural surface and were difficult to visualize with the naked eyes,^6)^ and their size and density made the sensation by finger touch not so obvious.^7)^ In addition, the incision of video-assisted thoracoscopic surgery (VATS) was small, while the fingers found it difficult to reach the nodules that were far away from the incision or deeper in the lung parenchyma. Unable to precisely locate the nodule may prolong the operation time, increase the probability of expanding the resection range, and even induce lesions residue and resection failure.^8)^ Therefore, a safe, reliable, and convenient method for nodule positioning was needed.

Currently, preoperative nodule positioning methods commonly used in clinical practice include the CT-guided hook^9)^/microcoil^10)^ implantation and percutaneous dyes/adhesives injection,^11)^ among others. However, these methods also induce risks such as pneumothorax, intrapulmonary hematoma, hook detachment/displacement, and embolism events.^12,13)^ Some novel positioning technologies that can be conducted in the operating room have been gradually applied in clinical practice, but they are not mature yet, and have high requirements for equipment and personnel, which limit their promotion and use.

At present, the technology of CT simulation positioning system used for radiotherapy is widely used. The system can accurately locate specific positions on the body surface corresponding to the CT image by assistance of auxiliary calibration in the laser system. On this basis, we designed a novel pulmonary nodule positioning technique. Through the simulation positioning system, the closest point on the body surface (surface puncture point) of the nodule’s pleural projection point is positioned before surgery. During the operation, fine needle thoracentesis is performed from the surface puncture point under the guidance of thoracoscopy to determine the pleural projection point of the nodule, so as to provide a clue for determining the extent of wedge resection. This method is easy to perform, can reduce pain in patients undergoing puncture and waiting while awake, and avoid the risks of common positioning techniques. This study tends to verify the efficacy and safety of this new method.

## Materials and Methods

### Ethical statement

The study was approved by the Hospital Ethics Committee of Zhejiang Cancer Hospital (Approval No.: IRB-2023-12) before initiation. All enrolled patients routinely signed the informed consent and agreed for publishing of relevant image data in medical publications.

### Inclusion and exclusion criteria

Inclusion criteria: (1) The age is ≥18 years and ≤80 years; (2) nodules are considered as early lung cancer (GGOs larger than 8 mm, or have an increasing trend in size or density during observation, with ratio of the solid component to the tumor diameter at ≤25%), benign tumors, oligometastatic tumors, and other lesions with wedge resection indications; (3) nodules are located in the outer third of the lung parenchyma; (4) the closest distance between the nodule edge and the pleura is ≥5 mm and ≤20 mm; and (5) the function of each organ can tolerate general anesthesia and lung surgery.

Exclusion criteria: (1) The nodule does not meet the indications for sublobar resection and lobectomy would be needed; (2) the nodule is close to the hilum or large blood vessels, and cannot be resected by wedge resection; (3) patients with severe pleural adhesion, which affect the process of positioning; (4) nodule that is technically impossible to be positioned by this method (the route from surface puncture point to pleural projection point is blocked by the scapula, or the nodule is close to the mediastinal surface); and (5) there are surgical/anesthesia contraindications such as heart/lung failure and taking anticoagulant drugs within the past 1 week.

### CT Simulation positioning process

All patients underwent chest thin-slice CT (1 mm) scan after admission, and completed other routine preoperative examinations. For patients who met the inclusion criteria, body surface positioning was performed under the CT simulation positioning system within 24 hours before surgery.

**Fig. 1:**
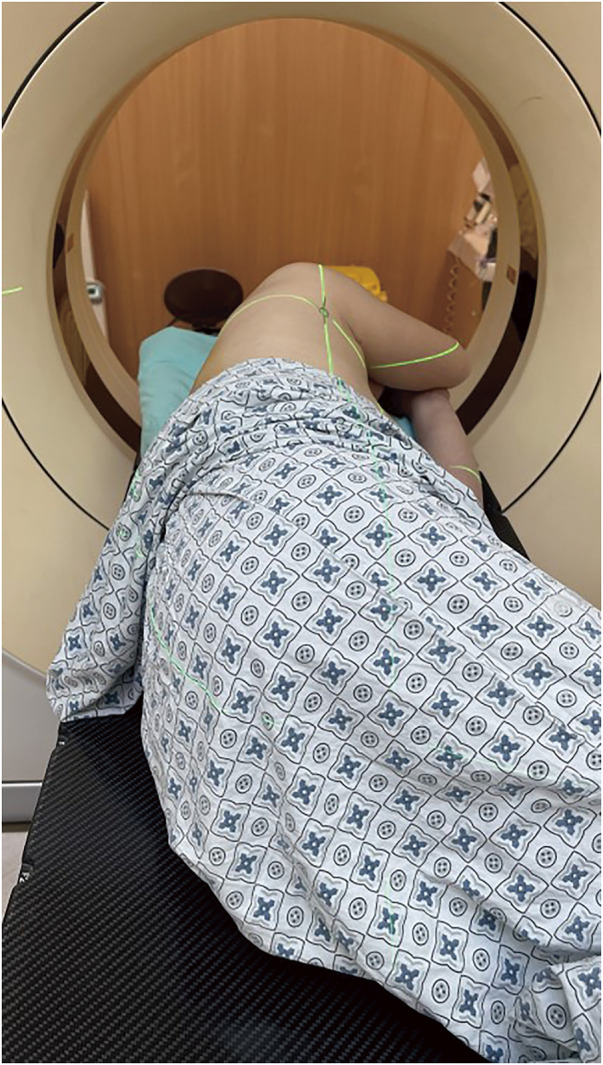
The patient lay on his side on the examination table of CT simulation positioning system, with both arms raised at a 90° angle (fully simulated surgical position).

**Fig. 2:**
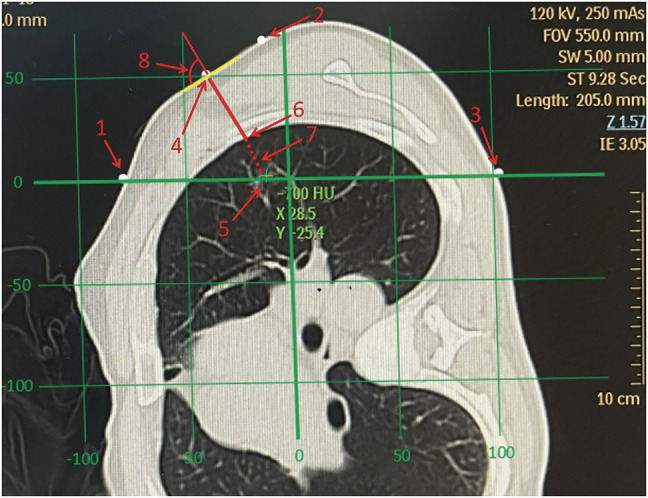
The positioning process under CT simulation positioning system. The numbers in the figure denote as follows: 1. Intersection point of horizontal line and patient’s ACW skin (intersection point of ACW); 2. Intersection point of vertical line and patient’s LCW skin (intersection point of LCW); 3. Intersection point of horizontal line and patient’s PCW skin (intersection point of PCW); 4. The closest point on the body surface to the pleural projection point of nodule (surface puncture point); 5. Target nodule; 6. Pleural projection point of nodule; 7. The connecting line between the lateral edge of the nodule to the pleural projection point; 8. The angle between connecting line (number 8) and the tangent line at the surface puncture point (puncture angle). ACW: anterior chest wall; LCW: lateral chest wall; PCW: posterior chest wall

**Fig. 3:**
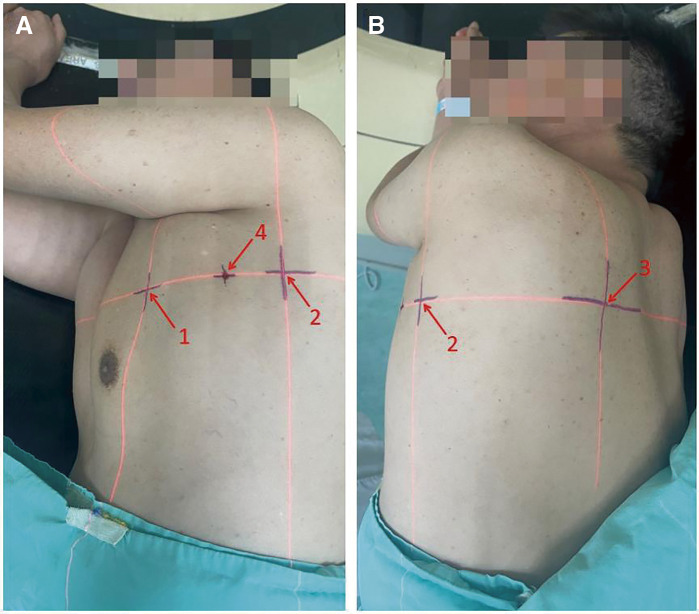
Skin markers after surface positioning process. The numbers in the figure denote as follows: 1. Intersection point of ACW; 2. Intersection point of LCW; 3. Intersection point of PCW; 4. Surface puncture point. ACW: anterior chest wall; LCW: lateral chest wall; PCW: posterior chest wall

The patient lay on his/her side on the CT simulation positioning system examination table, with both arms raised at a 90° angle (fully simulated surgical position) (**Fig. 1**). The system was turned on, with three sagittal laser lines and one coronal laser line intersecting on the body surface of the front chest wall, side chest wall, and back chest wall, respectively. The target nodule was scanned by thin-slice CT to determine that all the laser lines were located in the plane where the nodules are located. Next, the intersection of the horizontal laser line and the vertical laser line through the nodule and the body surface were marked. Then, the pleural projection point of the nodule (the closest point of the lateral edge of the nodule to the visceral pleura) was located on the CT image, and the connecting line was made between this point and the closest point on the body surface. The confirmed closest point on the body surface was the surface puncture point (**Fig. 2**). The angle between the connecting line and the tangent line at the surface puncture point was measured and used as the puncture angle. CT simulation positioning was performed again, and the specific position of the puncture point on the skin was marked with the assistance of Sure Mark (**Fig. 3**). After recording the angle between the connecting line and the tangent line, the thickness of the chest wall of the puncture path, and the distance between the lateral edge of the nodule and the visceral pleura, the process of CT simulation positioning was completed. All preoperative positioning procedures were performed by two surgeons from the same medical team with the assistance of radiation physicists who had at least 50 radiotherapy positioning experiences.

In addition, other concurrent positioning modalities such as CT-guided hook implantation were allowed.

### Intraoperative positioning process

After routine anesthesia induction, intubation, catheterization, and other preoperative procedures, the patient was placed in the lateral position (mimic the position of CT simulation positioning process), and routine disinfection and drape were performed.

**Fig. 4:**
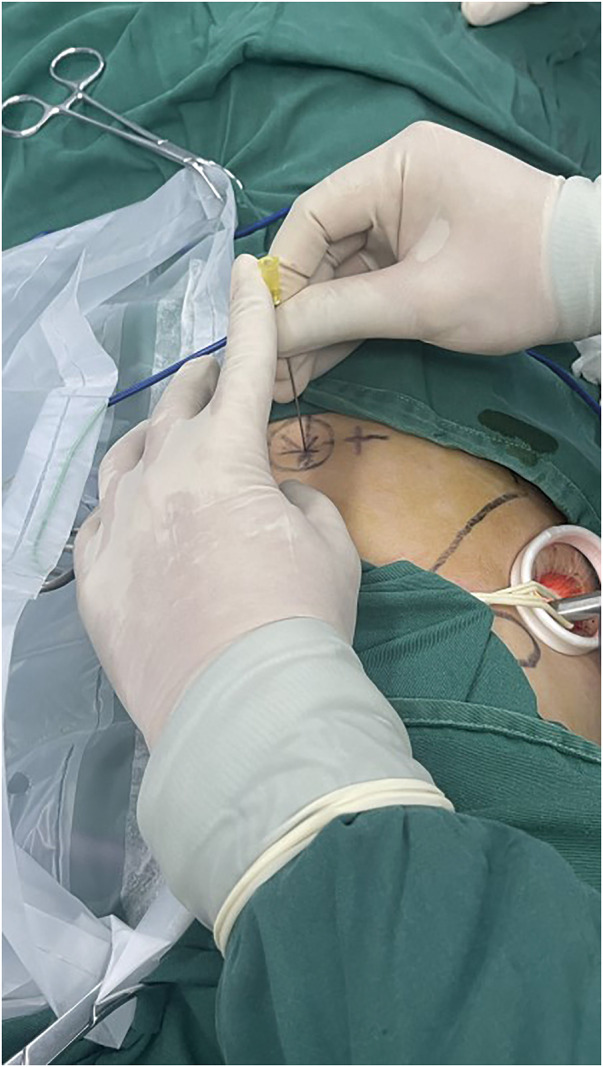
The position and angle of intraoperative puncture.

**Fig. 5:**
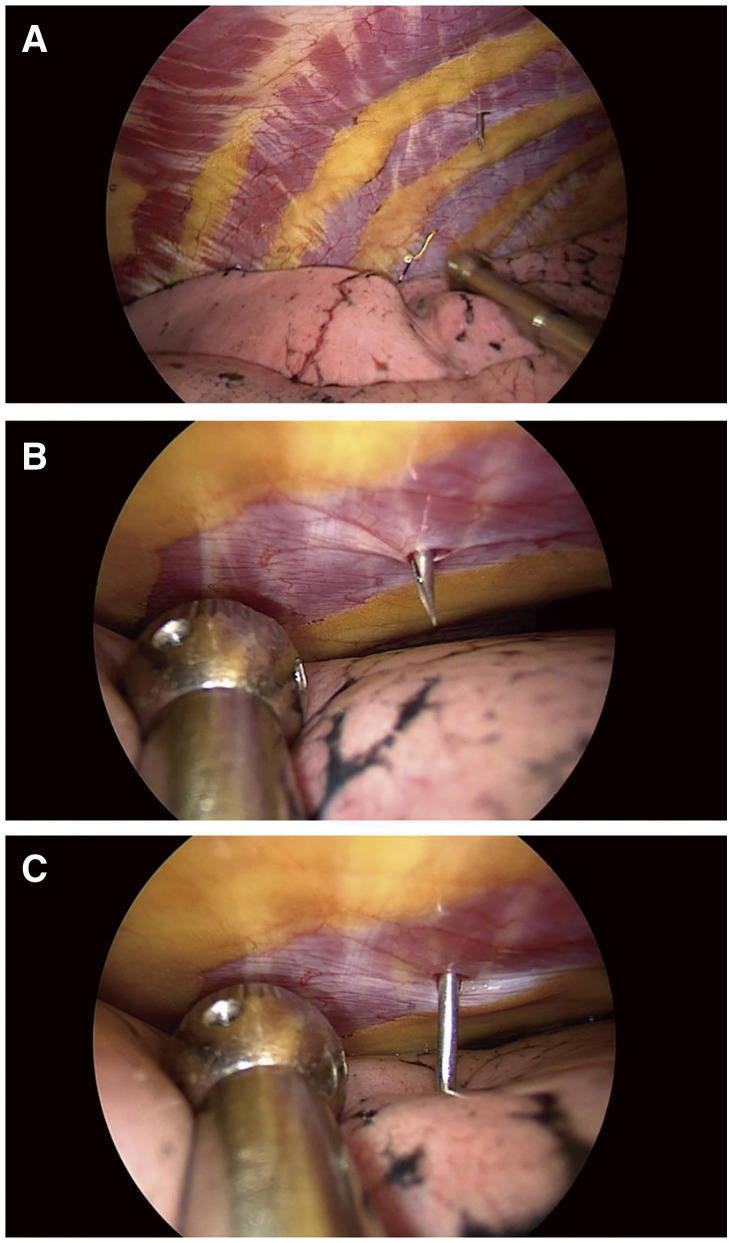
(**A**) The needle impaled parietal pleura, (**B**) The lung was fully inflated, (**C**) The needle penetrated into the visceral pleura by 0.5 cm.

**Fig. 6:**
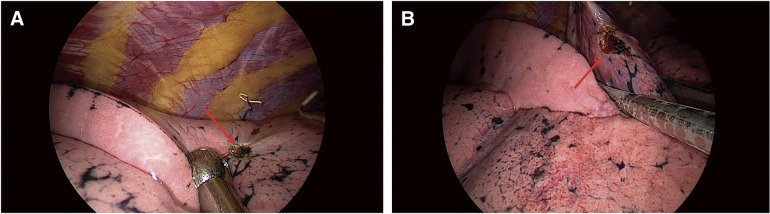
(**A**) The pinhole was burned with a condenser hook; (**B**) Wedge resection performed, including the burned mark region on visceral pleura.

The anesthesiologist performed one-lung ventilation of the healthy side. The incision was then made, and a thoracoscope was inserted. In our study, all patients underwent single port VATS. Under the guidance of thoracoscopy, a disposable needle was penetrated at the surface puncture point with the predetermined angle (**Fig. 4**), until the needle tip just broke through the parietal pleura. Then the anesthesiologist ventilated the affected lung. After the lung was fully inflated, the needle was pushed deeper until it penetrated the visceral pleura by 0.5 cm (**Fig. 5**). After one-lung ventilation was resumed on the healthy side, the lung on the affected side collapsed. The surgeon burned the pinhole with a condenser hook and left a conspicuous mark (**Fig. 6** and **Supplementary Video 1**). Finally, the needle was pulled out and the intraoperative positioning process was completed. During the puncture process, if the route of the needle is blocked by a rib, then the puncture is performed from the upper and lower edges of this rib along the vertical line, respectively. All intraoperative positioning processes were performed by two surgeons from the same medical team. The time spent from needle penetration to marking on the visceral pleura of the left lung was recorded.

### Resection process

A wedge resection was performed with a cutting stapler (combined with electric coagulation hook, ultrasonic knife, and other energy instruments when necessary) on lung tissue where the nodules were expected to be located. The resection range must include the burned mark region on the visceral pleura (**Fig. 6**). The resection depth must be larger than the distance between the lateral edge of the nodule and the visceral pleura and ensure a safe cutting edge (at least 10 mm).

**Fig. 7:**
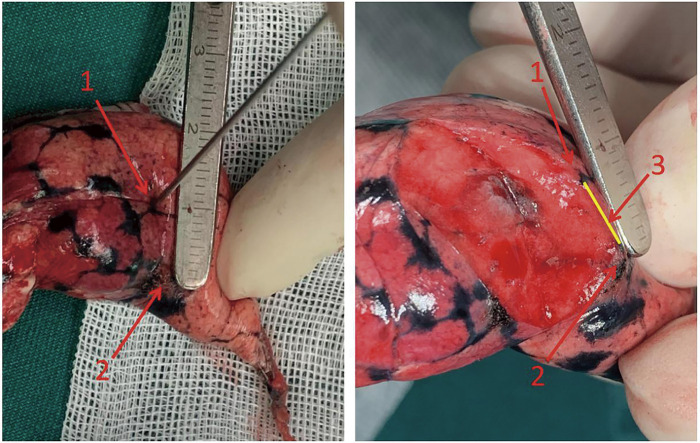
Measurement of positioning deviation distance. The numbers in the figure denote as follows: 1. Located the real pleural projection point of the nodule by touching; 2. The burned mark on visceral pleura; 3. Measure distance between the burned mark (pinhole) and the real pleural projection point, as positioning deviation distance.

After preparing the body, the surgeon locates the real pleural projection point of the nodule by touching. Then the resected lung tissue is cut open with an incision made along the line between the real projection point and the pinhole (burned mark), until the nodule is exposed. The distance between the real projection point and the burned mark (if there were two burned marks because of the rib block, the midpoint of the two points is taken as the exact burned mark) is measured, and this distance is defined as the positioning deviation distance (**Fig. 7**). The whole positioning process is considered successful if the small pulmonary nodule is completely resected and the positioning deviation distance is ≤20 mm. All resection and evaluation procedures were performed by two surgeons from the same medical team.

After completion of the procedure, the subsequent routine clinical principles are followed. The nodule is sent for intraoperative frozen pathological diagnosis, and then determined whether to expand the scope of resection or to terminate the operation directly.

### Statistical analysis

The data analysis is performed using SPSS v23.0. Two-sided *p* value <0.05 is regarded as the statistically significant difference. If the result of positioning deviation distance matches the normal distribution, variance analysis is used; if the result of positioning deviation distance does not match normal distribution, nonparametric test is used.

## Results

Between May 1, 2023 and May 30, 2024, a total of 73 patients who were expected to undergo a pulmonary wedge resection were screened and signed the informed consent. During the operation, one of them was found to have obvious adhesion in the area of the thoracic nodules, which was not suitable for intraoperative puncture positioning and withdrew from the study. Ultimately, 72 patients successfully completed the whole process of positioning and resection. Their average age was 53 years (27–76), 23 were male and the others were female; 13 cases had a family history of lung cancer, 12 cases had a long history of smoking, and all of them were male. A total of 85 nodules were resected in these 72 operations, and for each patient, at least one nodule was positioned and resected with our method; multiple nodules were also resected in the operation (**Table 1**). The average diameter of the target nodes was 9.6 ± 2.7 mm (5–16 mm).

**Table 1:** The baseline data of enrolled patients

Characteristics	Mean (SD)
Age (years)	53
Female (n, %)	68
Family history of lung cancer (n, %)	18.1
Male smoking rate (n, %)	52.2
Nodules average maximum size (mm)	9.6 ± 2.7
Average positioning deviation distance (mm)	8.1 ± 4.4
Average positioning deviation distance for nodules blocked by ribs (mm)	13.8 ± 7.2
The average positioning time (s)	191

The intraoperative positioning process took an average of 191 ± 62.1 seconds (77–357 seconds). All patients’ nodules were successfully resected, and 70 patients’ pulmonary nodules were successfully located by this method (positioning deviation distance ≤20 mm). The average positioning deviation distance was 8.1 ± 4.4 mm (0–27 mm), and the accuracy of positioning was 97.2%. For 6 patients, the needle route was blocked by the ribs and punctures were performed from the upper and lower edges of this rib along the vertical line, respectively; their average positioning deviation was 13.8 ± 7.2 mm. There were no related complications such as bleeding and hematoma.

According to the position of needle penetrating point, the area before the anterior axillary line was defined as the anterior chest wall (ACW), the area between the anterior axillary line and the posterior axillary line was defined as the lateral chest wall (LCW), and the area after the posterior axillary line was defined as the posterior chest wall (PCW). We performed subgroup analysis according to the nodules’ locations and needle penetrating directions. The positioning deviation distance of nodules in the different lung lobes differed, as shown in **Table 2**. The right upper/middle lobe and the left upper lobe had a higher proportion of ACW punctures, with more nodules positioning deviation distance >10 mm, while the right lower lobe and the left lower lobe had a higher proportion of PCW and positioning deviation distance ≤10 mm. For nodules in the right upper/middle lobe and the left upper lobe, the positioning deviation distances were significantly larger (*p* ≤0.05), as shown in **Table 3**. The average positioning deviation distance of puncture directions is shown in **Table 4**. The average positioning deviation distance of the PCW was 5.4 ± 2.3 mm, which was significantly smaller than that of the ACW (9.2 ± 3.4 mm) and LCW (9.6 ± 5.5 mm); the difference is statistically significant at *p* ≤0.05.

**Table 2:** Distribution of puncture directions and positioning deviation distance for nodules located in different lobes

Lobe	Deviation distance (mm)	ACW	LCW	PCW	Total	Mean ± SD (mm)
RUL	0–10	5	4	1	10	10.25 ± 5.7
	11–20	3	2	0	5	
	>20	0	1	0	1	
RML	0–10	6	3	0	9	10.1 ± 4.6
	11–20	1	1	0	2	
	>20	0	1	0	1	
RLL	0–10	0	1	8	9	6.3 ± 2.7
	11–20	0	1	0	1	
	>20	0	0	0	0	
LUL	0–10	2	3	1	6	9.5 ± 3.4
	11–20	4	1	0	5	
	>20	0	0	0	0	
LLL	0–10	1	7	14	22	5.6 ± 2.5
	11–20	0	0	1	1	
	>20	0	0	0	0	

Statistical ratios of different lung lobes: RUL:RLL, *p* = 0.035; RML:RLL, *p* = 0.023; RUL:RML, *p* = 0.747; LUL:LLL, *p* = 0.002.

ACW: anterior chest wall; LCW: lateral chest wall; PCW: posterior chest wall; RUL: right upper lobe; RML: right middle lobe; RLL: right lower lobe; LUL: left upper lobe; LLL: left lower lobe

**Table 3:** Nodules in the RUL, RML and LUL had a larger positioning deviation distance than those in RLL and LLL

Deviation distance (mm)	RUL + RML + LUL	RLL + LLL
0–10	25	31
11–20	12	2
>20	2	0
Total	39	33
Mean, mm (SD)	10.0 (4.7)	5.8 (2.6)

Statistical ratios of different lung lobes: (RUL + RML + LUL):​(RLL + LLL), *p* <0.001

**Table 4:** The average positioning deviation distance of different puncture directions

The puncture location	0–10 mm (n)	11–20 mm (n)	>20 mm (n)	Average positioning deviation distance
ACW	14	8	0	9.2 (3.4)
LCW	18	5	2	9.6 (5.5)
PCW	24	1	0	5.4 (2.3)

Statistical ratio of the mean positioning deviation distance of needle entry from different regions: ACW:LCW, *p* = 0.861; ACW:PCW, *p* <0.001; LCW:PCW, *p* <0.001.

ACW: anterior chest wall; LCW: lateral chest wall; PCW: posterior chest wall

## Discussion

With a significant increase in the incidence of early lung cancer such as GGOs, wedge resection became more and more common, and the demand for accurate positioning of nodules also increased. Nodule positioning methods commonly used can be divided into two categories: preoperative positioning and intraoperative positioning. The former is generally performed outside the operating room, and the patient is in the awake state during the positioning process. The latter is performed in the operating room with the assistance of some instruments and equipment.

Preoperative positioning is more commonly used. CT-guided hook/coil positioning is the representative method, which is considered to be the most reliable positioning technique, and is safe and effective even when the nodule is smaller than 10 mm. However, the hook might fall off and get displaced, and the incidence of postoperative pneumothorax would be significantly increased when multiple nodules are positioned at the same time. In addition, the incidence of hook/coil falling off would increase significantly when the nodule is near the visceral pleura.^14)^

Preoperative positioning with methylene blue is the most common method of dye positioning. Compared with hook/coil positioning, being simple and cheap are its biggest advantages. However, injection of too much dye and too long an interval time after injection can easily lead to dye diffusion. It is necessary to perform surgical resection quickly after the positioning is completed.^15)^ A study showed that the time for methylene blue staining could be extended from 2 to 22 hours by mixing methylene blue with autologous blood, and a clear blue-purple blood spot can be seen during the operation.^16)^ However, when locating deeper nodules, the injected methylene blue might be difficult to be seen on the surface of the visceral pleura.

Most of the preoperative positioning methods had similar limitations, including additional invasive operation, preoperative complications, extra pain, and psychological pressure. If some conditions (e.g., arrhythmia during induction of anesthesia) for obstructing surgery happened after positioning was completed but before the operation started, the implanted device became even more problematic. These limitations led to more innovation.

Intraoperative ultrasound positioning is one of the first reported intraoperative positioning methods. The success rate of ultrasound for detection of pulmonary nodules is as high as 93%, and most of the ultrasonographic measured margins are consistent with the actual histological margins. Noninvasive and high repeatability are the main advantages of ultrasonic positioning, but this method requires the lung to achieve adequate collapse. For patients with obvious emphysema, the residual air in the lung tissue produces a large number of inflation artifacts on the ultrasound image, which would affect the positioning.^17,18)^ Electromagnetic navigation bronchoscopy (ENB) dye positioning technology is based on the preoperative bronchoscope path simulation; it guides the bronchoscope and injection catheter into the bronchiole around the nodule under electromagnetic field, and then dye injection is performed. However, the equipment required in the operating room is expensive, and the time for surgeons to learn is long.^19,20)^ Another study reported intraoperative electromagnetic transthoracic nodule localization (EMTTNL) technology. This technology used ENB to identify the target nodule’s position, punctured with a needle from the body surface through a planned route, and injected dye to the target nodule. This method is suitable for small and deep nodules, but the use of methylene blue would lead to excessive diffusion of staining on the pleura, which might induce extended segmentectomy.^21)^

With the development of technology, virtual reality is being used in the positioning of lung nodules. Augmented reality navigation guidance is based on 3-dimensional (3D) virtual images constructed by CT images, which develops a personalized preoperative puncture plan for the patient. After entering the anesthesia state, the doctor performs a puncture with the help of head-mounted augmented reality equipment. Most of the work can be completed before operation, and the procedures in the operation room are relatively simple. However, 30% of the patients in the previous study failed due to software problems and operation mistakes, and the technology remained unstable.^22)^

The positioning method introduced in this study is a novel technique in which preoperative body surface positioning is performed under the CT simulation positioning system, followed by intraoperative puncture. The two steps of positioning and puncture are separate. Our study confirmed that the success rate of this method was 97.2%, which was satisfactory. Compared with traditional methods of preoperative positioning, the positioning method has unique advantages. First, the preoperative positioning process is completely noninvasive, and the only invasive operation is performed after anesthesia to avoid pain and tension. Second, the puncture process is completely under the guidance of thoracoscopy, which will not damage the intercostal blood vessels and nerves, leave alone pneumothorax, intrapulmonary hematoma, air embolism, and other serious complications. Third, no items are planted preoperative, which means the patients are able to cancel the operation anytime before anesthesia, subjectively or passively. Fourth, the average time of positioning puncture during the operation was only 191 seconds, which means the method is convenient, and will not cause significant prolongation of operation and anesthesia. Finally, this positioning method did not require additional equipment and is convenient for doctors to acquire. Patients only need to pay the cost of radiotherapy positioning (less than 100 yuan, which could also enjoy the reimbursement policy of medical insurance), which was significantly cheaper than the cost of traditional CT-guided hook/coil positioning.

Zhao et al.^23)^ used preoperative high-resolution CT scan to determine the precise positional relationship between the nodule and the nearest rib, and determine the specific position of nodule projection point on the body surface with a help of a dial. This idea was similar to our positioning method. The essential difference was that we use the laser positioning system to carry out the projection correspondence between the CT image and the specific position on the patient’s body surface, which is more accurate. After determining the point on the body surface, another CT scan can be performed immediately to confirm the position. Moreover, under the CT simulation positioning system, the patient can take the same lateral position as in the operation room, to avoid deviation of the positioning point caused by the change in body position.

In the subgroup analysis, we found that the mean positioning deviation distance was shorter when the needle was inserted through the PCW, compared with ACW and LCW. On the one hand, although the patient also took the surgical position during the simulation positioning process, it could not be exactly the same as the position in the real operation, and the skin of the ACW and LCW was more susceptible to postural changes. On the other hand, the PCW has thinner chest tissue and less deviation during needle puncture. We also found that the positioning deviation distance for nodules of the right upper/middle lobes and the left upper lobe was much further. The reason might be that the area covered by the scapula mainly belonged to these lobes, and the proportion of puncture from PCW was lower, which affected the average positioning deviation distance. In addition, it is worth mentioning that this method required the needle puncture along the measured angle; after measurement in practical operation, we found the angle to be approximately vertical (average greater than 87°), which means it is more conducive for clinical operation.

Although the positioning method is simple and feasible, there are still some limitations. First, the method is not suitable for nodules occluded by bony structures (e.g., scapula, clavicle), while for nodules beneath the ribs, this method is also relatively inaccurate. In addition, for pulmonary nodules located deep or close to the mediastinal surface, the relative position between the pleural projection points and the nodule changes greatly during the process of lung inflation–collapse, so the use of this method needs to be cautious. Third, the posture during the CT simulation positioning process and during operation may show some differences, which may influence the accuracy of positioning. Furthermore, this method requires the surgeon to handle the angle of puncture, especially for patients with a thick chest wall, or the positioning point close to the axilla (where the chest wall tissue is the thickest under the operation position), where a small deviation in the puncture angle may have a great influence on the positioning deviation distance. In this study, the two nodules with the positioning deviation distance larger than 20 mm (defined as failed positioning) were both in this situation.

This study was a single center, single-arm study. The sample quantity was not large, and direct comparison between clinical commonly used methods such as CT-guided hook/coil positioning was not performed. Further large-scale randomized controlled trials were needed to evaluate and verify the safety and efficacy of this technique.

## Conclusion

The results of this study show that the body surface positioning method of lung nodules under CT simulation positioning system has a high success rate, high positioning accuracy, good safety, strong applicability and cheap cost, which are worthy of clinical application.

## Declarations

### Ethics approval and consent to participate

The study was approved by the Hospital Ethics Committee of Zhejiang Cancer Hospital (Approval No.: IRB-2023-12).

### Consent for publication

Written informed consent for publication was obtained from all participants.

### Funding

The study is sponsored by the Major Projects Jointly Constructed by Zhejiang Traditional Chinese Medicine (GZY-ZJ-KJ-23004), National Key Scientific Program of China (2022YFA1304500), and Zhejiang Medicine and Health Plan Project (2022KY082).

### Data availability

The data that support the findings of this study are available on request from the corresponding author, Jian Zeng, upon reasonable request.

### Authors’ contributions

(1) Conception and design: Jiandong Hong, Taobo Luo and Jian Zeng; (2) Administrative support: Jian Zeng; (3) Provision of study materials or patients: Jian Zeng; (4) Collection and assembly of data: Jiandong Hong, Yan Zhang, Ying Chen, Yang Pan, and Haoting Xu; (5) Data analysis and interpretation: Jiandong Hong, Yan Zhang, Taobo Luo, and Jian Zeng; (6) Manuscript writing: all authors; (7) Final approval of manuscript: all authors.

### Disclosure statement

All authors have no conflicts of interest to declare.

## Supplementary Material

Video 1. The process of intraoperative positioning. After the lung was fully inflated, the needle was pushed deeper until it penetrated the visceral pleura by 0.5 cm; after the lung on the affected side collapsed, the surgeon burned the pinhole with a condenser hook, and left a conspicuous mark.

## References

[1] Lancaster HL Heuvelmans MA Oudkerk M. Low-dose computed tomography lung cancer screening: clinical evidence and implementation research. J Intern Med 2022; 292: 68–80. 35253286 10.1111/joim.13480 PMC9311401

[2] Henschke CI Yip R Smith JP Ct screening for lung cancer: part-solid nodules in baseline and annual repeat rounds. AJR Am J Roentgenol 2016; 207: 1176–84. 27726410 10.2214/AJR.16.16043

[3] Walter JE Heuvelmans MA Yousaf-Khan U New subsolid pulmonary nodules in lung cancer screening: the NELSON trial. J Thorac Oncol 2018; 13: 1410–4. 29775805 10.1016/j.jtho.2018.05.006

[4] Sato M. Precise sublobar lung resection for small pulmonary nodules: localization and beyond. Gen Thorac Cardiovasc Surg 2020; 68: 684–91. 31654291 10.1007/s11748-019-01232-1

[5] Suzuki K Watanabe SI Wakabayashi M A single-arm study of sublobar resection for ground-glass opacity dominant peripheral lung cancer. J Thorac Cardiovasc Surg 2022; 163: 289–301.e2. 33487427 10.1016/j.jtcvs.2020.09.146

[6] Demmy TL Wagner-Mann CC James MA Feasibility of mathematical models to predict success in video-assisted thoracic surgery lung nodule excision. Am J Surg 1997; 174: 20–3. 9240946 10.1016/S0002-9610(97)00021-4

[7] Thistlethwaite PA Gower JR Hernandez M Needle localization of small pulmonary nodules: lessons learned. J Thorac Cardiovasc Surg 2018; 155: 2140–7. 29455962 10.1016/j.jtcvs.2018.01.007

[8] Seo JM Lee HY Kim HK Factors determining successful computed tomography-guided localization of lung nodules. J Thorac Cardiovasc Surg 2012; 143: 809–14. 22104686 10.1016/j.jtcvs.2011.10.038

[9] Park CH Lee SM Lee JW Hook-wire localization versus lipiodol localization for patients with pulmonary lesions having ground-glass opacity. J Thorac Cardiovasc Surg 2020; 159: 1571–9. 31735392 10.1016/j.jtcvs.2019.08.100

[10] Yu S Li W Liu X Application value of CT-guided localization using a coil in combination with medical adhesive in sublobar resection. Clin Transl Oncol 2023; 25: 2931–7. 37020165 10.1007/s12094-023-03156-y

[11] Wang L Sun D Gao M Computed tomography-​guided localization of pulmonary nodules prior to thoracoscopic surgery. Thorac Cancer 2023; 14: 119–26. 36482812 10.1111/1759-7714.14754 PMC9834693

[12] Zhang H Li Y Yimin N CT-guided hook-wire localization of malignant pulmonary nodules for video assisted thoracoscopic surgery. J Cardiothorac Surg 2020; 15: 307. 33036640 10.1186/s13019-020-01279-9 PMC7545541

[13] Yao F Wang J Yao J Reevaluation of the efficacy of preoperative computed tomography-guided hook wire localization: a retrospective analysis. Int J Surg 2018; 51: 24–30. 29367030 10.1016/j.ijsu.2018.01.014

[14] Zuo T Gao Z Zhang T Preoperative small pulmonary nodule localisation using hookwires or coils: strategy selection in adverse events. J Cardiothorac Surg 2023; 18: 237. 37488567 10.1186/s13019-023-02301-6 PMC10367412

[15] Zhang SF Liu HR Ma AL Preoperative computed tomography-guided localization for multiple pulmonary nodules: comparison of methylene blue and coil. J Cardiothorac Surg 2022; 17: 186. 35986299 10.1186/s13019-022-01941-4 PMC9389799

[16] Feng Z Liao QX Xie JB Utility of methylene blue mixed with autologous blood in preoperative localization of pulmonary nodules and masses. Open Life Sci 2023; 18: 20220645. 37465103 10.1515/biol-2022-0645 PMC10350882

[17] Khereba M Ferraro P Duranceau A Thoracoscopic localization of intraparenchymal pulmonary nodules using direct intracavitary thoracoscopic ultrasonography prevents conversion of VATS procedures to thoracotomy in selected patients. J Thorac Cardiovasc Surg 2012; 144: 1160–5. 22980667 10.1016/j.jtcvs.2012.08.034

[18] Taurchini M Quarato CMI Frongillo EM Intraoperative lung ultrasound (ILU) for the assessment of pulmonary nodules. Diagnostics (Basel) 2021; 11: 1691. 34574032 10.3390/diagnostics11091691 PMC8466360

[19] Han KN Kim HK. The feasibility of electromagnetic navigational bronchoscopic localization with fluorescence and radiocontrast dyes for video-assisted thoracoscopic surgery resection. J Thorac Dis 2018; 10(Suppl 6): S739–48. 29732195 10.21037/jtd.2018.03.115 PMC5911741

[20] Song JW Park IK Bae SY Electromagnetic navigation bronchoscopy-guided dye marking for localization of pulmonary nodules. Ann Thorac Surg 2022; 113: 1663–9. 34052219 10.1016/j.athoracsur.2021.05.004

[21] Long J Petrov R Haithcock B Electromagnetic transthoracic nodule localization for minimally invasive pulmonary resection. Ann Thorac Surg 2019; 108: 1528–34. 31233723 10.1016/j.athoracsur.2019.04.107

[22] Li C Ji A Jian Z Augmented reality navigation-guided intraoperative pulmonary nodule localization: a pilot study. Transl Lung Cancer Res 2023; 12: 1728–37. 37691871 10.21037/tlcr-23-201 PMC10483087

[23] Zhao L Yang W Hong R Application of three-dimensional reconstruction combined with dial positioning in small pulmonary nodules surgery. J Cardiothorac Surg 2021; 16: 254. 34496890 10.1186/s13019-021-01642-4 PMC8424933

